# A gut feeling of statin

**DOI:** 10.1080/19490976.2024.2415487

**Published:** 2024-10-29

**Authors:** Jianqing She, Lizhe Sun, Yue Yu, Heze Fan, Xia Li, Xinyu Zhang, Xiaozhen Zhuo, Manyun Guo, Junhui Liu, Peining Liu, Gulinigaer Tuerhongjiang, Bin Du, Hongbing Li, Jun Yu, Zuyi Yuan, Yue Wu

**Affiliations:** a Cardiovascular Department, First Affiliated Hospital of Xi’an Jiaotong University, Xi’an, Shaanxi, China; b Key Laboratory of Environment and Genes Related to Diseases, Ministry of Education, Xi’an, Shaanxi, China; c Cardiometabolic Innovation Center, Ministry of Education, Xi’an, Shaanxi, China; d Clinical Laboratory, First Affiliated Hospital of Xi’an Jiaotong University, Xi’an, Shaanxi, China; e Department of Medicine and Therapeutics and Institute of Digestive Disease, The State Key Laboratory of Digestive Disease, The Chinese University of Hong Kong, Hong Kong SAR, China

**Keywords:** Statins, gut microbiota, drug metabolism, cardiovascular health, personalized treatment

## Abstract

Statins, known as HMG-CoA reductase inhibitors, are widely utilized to reduce blood cholesterol levels and possess pleiotropic effects, including the influence on inflammation and macrophage proliferation. Despite their significant impact in diminishing the incidence of cardiovascular events and mortality, individual responses to statin therapy vary considerably. Understanding this variability is essential for optimizing treatment outcomes and minimizing adverse effects. The gut microbiota, a complex ecosystem of microorganisms within the gastrointestinal tract, plays a critical role in human health and disease. Emerging evidence has linked the gut microbiota to drug metabolism and response, with the potential to modulate the efficacy of statin therapy and its side effects. This review provides a comprehensive overview of the interaction between the gut microbiota and statins. It discusses how the gut microbiota can influence the therapeutic effects and side effects of statins and examines the mechanisms by which the gut microbiota affect statin response and cardiovascular diseases.

## 1. Introduction

Statins, known as 3-hydroxy-3-methylglutaryl coenzyme A-reductase inhibitors, are widely used to lower cholesterol levels in the blood and exert pleiotropic effects, such as inhibition of inflammation and macrophage proliferation. By reducing cholesterol production, statins help lower LDL cholesterol and triglycerides levels, while also increasing HDL cholesterol levels.^1,2^ Numerous studies have shown that statins can significantly decrease the incidence of cardiovascular events and mortality in patients with or at risk of cardiovascular disease.^3–5^ Extensive researches have discussed potential effects of statin on various health conditions beyond cardiovascular disease. Some studies suggest that statins may have a protective effect on cognitive decline in patients with Alzheimer’s and mixed dementia, potentially due to their multifaceted actions on cholesterol-dependent and independent pathways that influence neurodegenerative processes.^6^ A nationwide Swedish cohort study has also indicated that statin exposure may reduce the risk of severe COVID-19 outcomes, including hospitalization and death.^7–9^ The protective effects of statins could be attributed to their anti-inflammatory effects, reduction of reactive oxygen species, and lipid regulatory effects, which may modulate the host response among COVID-19 patients. In addition, statins have also been proposed as a potential treatment for acute sarcopenia, a condition characterized by the loss of muscle mass and function, especially in older adults.^10^ However, there is considerable variation in how individuals respond to statin therapy. Many individuals may not derive the same benefits as others or encounter intolerable side effects, despite adherence to recommended statin therapy.^11,12^ Understanding the variability in individual responses to statin therapy is crucial for optimizing outcomes and minimizing adverse effects.

The gut microbiota, a complex ecosystem of microorganisms residing in the gastrointestinal tract, has emerged as a crucial player in human health and diseases. It has been estimated that approximately 40 trillion microorganisms constitute the human gut microbiota.^13^ The gut microbial gene set is 150 times greater than the human genome.^14^ Although individual differences exist, the common phyla are *Firmicutes* and *Bacteroidetes* (90% of the population), followed by *Actinobacteria* and *Verrucomicrobia* in healthy population.^15^ Intestinal flora is regulated by multiple endogenous and exogenous determinants, such as individual variation, diet, and exercise.^16^ Disturbance in the composition and diversity of gut microbiome, often called dysbiosis, plays a pivotal role in the pathogenesis of several diseases such as cardiovascular diseases (hypertension, atherosclerosis, and heart failure),^13,17–19^ metabolic diseases (obesity, hyperlipidemia, and diabetes),^20–22^ and gastrointestinal diseases (inflammatory bowel disease and colorectal cancer).^23,24^ Targeting the gut microbiome may provide a potential therapeutic approach in the treatment of these diseases.

Recently, increasing evidence has linked the gut microbiota to drug metabolism and response. Gut microbiota play a critical role in host physiology and pathology. On the one hand, gut microbiota play key immunological, homeostatic and metabolic functions in the human health.^16^ On the other hand, the intestinal flora disorder is associated with many diseases such as cardiovascular diseases, diabetes, obesity, cancer and so on. ^25,26^ Rapid advances in sequencing technologies and bioinformatics have deeply promoted our cognition on the connection between gut microbiota and drugs. Numbers studies have proved that drugs could modulate intestinal flora and affect the host’s response.^12,25,27^ Medications, like statin, may impact the composition, abundance and diversity of the gut microbiota and alter the normal interactions between microbiota and the host.^28^ Reversely, the microbiota may result in altered drug pharmacokinetics and pharmacodynamics by metabolizing medications or disturbed drug efficacy by producing toxic metabolites. Manipulating gut microbiota may be a promising target to enhance the effectiveness of statin therapy. Understanding how the gut microbiota interact with statins will provide valuable insights into optimizing treatment outcomes, minimizing side effects, and developing novel precision therapeutics for cardiovascular diseases.

**Figure 1.**
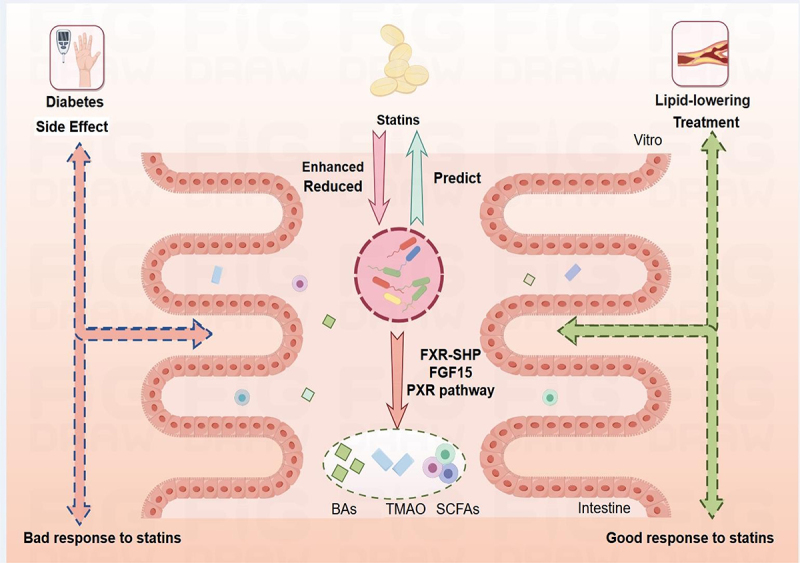
The overview of statins in gut microbiota.

In this review, we provide a comprehensive overview of gut microbiota–statin interaction (Figure 1). We discuss how gut microbiota modulate therapeutic and side effects of statin, and examine the mechanisms by which the microbiome exerts its effects on statin response and cardiovascular diseases.

Statins enter the intestine and affect the abundance of gut microbiota; meanwhile, the gut microbiota could predict statins response or adverse events. Statins could produce secondary metabolites (TMAO/BAs/SCFAs) by different mechanisms. Gut microbiota play a key role in the treatment and side effects of statins. Finally, statins-induced changes of gut microbiota are associated with individual differences. BA: bile acid, FGF15: fibroblast growth factor 15, FXR-SHP: farnesoid X receptor-small heterodimer partner, PXR: pregnane X receptor, SCFAs: short-Chain Fatty Acids, TMAO: trimethylamine oxide. Created by Figdraw.

## 2. Impact of statins on gut microbiota composition

A lot of researches have revealed alterations in the composition and function of gut microbiota in patients taking different statins.^12,29–45^ Besides the bacterial species, diversity and abundance of gut microbiota are influenced by this remodeling. A growing number of evidence demonstrates that the treatment of statins could directly or indirectly affect the diversity and abundance of gut microbiota. Statins can directly increase the relative abundance of Bacteroidetes and decrease the abundance of Firmicutes.^42,43^ Caparrós-Martín et al.^46^ used a 16S rRNA gene sequencing approach to explore the changes in three different treatments (pravastatin, atorvastatin, or no treatment) in the murine model. It is identified that statin therapy altered gut microbiota in mice through a pregnane X receptor (PXR)-dependent mechanism.^46^ Besides, *Clostridium*^12^ and *Akkermansia muciniphila*^30^ were directly decreased by statin treatment. In contrast, a study examining fecal samples from 5755 individuals in the FINRISK-2002 population cohort found that the usage of statin is associated with the changes of the gut microbiota, including differing taxonomic composition and 13 differentially abundant species in fully adjusted models.^47^ Furthermore, growing studies proved that statin usage correlates to the enhancement of the phylum *Verrucomicrobia*,^37^ and the genera *Bacteroides*, including *Butyricimonas, Mucispirillum,*^33^ *Oscillospira*,^32^ *Akkermansia, Lactobacillus,*^37^ *Bifidobacterium*, *Anaerostipes, Ruminococcus*,^28^ *Eubacterium,* and *Faecalibacterium.*^48^ In addition, the statin therapy was also associated with decreased abundance of the genera *Desulfovibrio*^32^ and *Parabacteroides.*^28^ Interestingly, the cholesterol-lowering and other regulatory effects of statins were significantly attenuated or altered upon depletion of gut microbiota.^42^

A number of pathways and mechanisms related to the interaction between statin and gut microbiota have been investigated. Inflammation plays key roles in this progress. Statin could reduce the gut microbiota, which is associated with inflammation. *Bacteroides2* (Bact2) enterotype, an intestinal microbiota configuration related to systemic inflammation, has a high prevalence in obese participants.^35^ This study also found that obesity-associated microbiota dysbiosis is negatively associated with statin treatment, resulting in a lower Bact2 prevalence of 5.88% in statin-medicated obese participants.^35^ In addition, Kim et al. found that statin treatment could increase the expression of the TGFβ-1, and decrease IL-1β.^33^ Meanwhile, they also found the expression level of TGFβ1 was positively correlated with the abundance of one of the gut microbiota, *Dorea,* while the expression level of IL-1β was negatively correlated the abundance of Mucispirillum in the statin group.^33^ These evidences suggest inflammation could be a confounder in the reaction between statin and gut microbiota. However, there are different sounds here. Cheng et al. found that atorvastatin could upregulate the colon p65, the key marker of activation of Nuclear Factor-kappa B (NF-κB) signaling pathway, and downregulate the expression of anti- inflammation genes, such as Hspa1a, Egr1^30^. These data perhaps provide a new view that statin could activate the NF-κB pathway, even leading to intestinal chronic inflammation.^30^ In addition, Nolan et al reported that statins directly inhibited the expression of systemic inflammatory markers in statin-treated animals, including TNF-α, IL-1β, and NF-κB.^49^ Interestingly, they also observed a significant increased expression of genes encoding TNF-α, TGF-β, and IL-1β in the ileum of statin-treated animals.^49^ These findings highlight the complexity of inflammation in the relationship between statin and gut microbiota. More studies are needed to confirm the role of inflammation in statin and gut microbiota.

In recent years, more and more studies have focused on the interaction of statins and circulating metabolites by regulating gut microbiota, and its influence on the therapeutic effects and side effects of statin.

## 3. Effect of statins on secondary metabolism

The gut microbiota could modulate gastrointestinal metabolism by affecting the synthesis, digestion, fermentation, and secondary metabolism of gut metabolites. Secondary metabolites play critical roles in drug metabolism and gut microbiota. The common secondary metabolites are trimethylamine/trimethylamine N-oxide (TMA/TMAO), short-chain fatty acids (SCFAs), and bile acids (BAs). TMA comes from the metabolism of choline-containing compounds by the gut microbiota, such as choline, betaine, and L-carnitine.^50^ Then, TMA is oxidized to produce TMAO in liver.^51^ It have been found that TMAO supplementation promoted the expression of multiple macrophage scavenger receptors linked to atherosclerosis, and the elevated level of TMAO could be a predictor of cardiovascular diseases.^52^ SCFAs, a subset of carboxylic acids with carbon atom numbers less than 6, are metabolized by the specific gut microbiota fermenting cellulose and resistant starch in the cecum and colon.^53,54^ The most common SCFAs are acetate, propionate and butyrate. SCFAs have a potentially beneficial role in preventing cardio-metabolic diseases through the improvement of the gut barrier, regulation of the blood pressure, modulation of glucose and lipid metabolism, mediation of the immune system and anti-inflammatory response.^16^ BAs are amphipathic steroid acids that come from cholesterol in the liver which are divided into two types: primary and secondary.^55^ The primary BAs are synthesized by the catabolism of cholesterol in the liver, and the secondary BAs are metabolized from primary BAs by the gut microbiota.^56^ BAs influence the host’s health by regulating the immune system and inflammation or the secondary BAs, such as ursodeoxycholic acid (UDCA).^12,57^

Growing numbers of studies have proved that imbalanced metabolites are correlated with diseases. TMAO metabolized by the gut microbiota is an atherogenic metabolite.^58,59^ Elevated TMAO is associated with an increased risk of cardiovascular disease, such as abdominal aortic aneurysm (AAA) and thrombosis.^60–62^ TMAO also promotes the occurrence of other diseases, such as diabetes, chronic kidney disease, cancer, non-alcoholic fatty liver disease, neurodegenerative disease, and obstructive sleep apnea.^63–66^ Generally, SCFAs are considered to be beneficial for human health and the supplementation of SCFAs could improve the host’s immunity.^16^ For example, the increase of microbiota-derived metabolite propionic acid (PA) may reduce the intestinal cholesterol absorption and atherosclerotic lesion area through upregulated regulatory T-cell numbers and interleukin-10 levels and downregulated intestinal cholesterol transporter – Niemann-Pick C1-like 1 level.^67^ Besides, the acetate supplementation significantly reduced the risk of hypertension and heart failure in hypertensive mice by suppressing the cardiac and renal Egr1, a key cardiovascular factor involved in cardiac hypertrophy, cardiorenal fibrosis, and inflammation.^68^ BAs are connected with metabolic syndromes, such as nonalcoholic steatohepatitis, obesity, inflammatory bowel disease, and colorectal cancer.^69–71^ Disturbed BA homeostasis is a key risk factor for metabolic, inflammatory, infectious, and tumorous diseases. The treatments targeting BAs and the microbiota have succeeded in some diseases. For example, the supplement of UDCA has been shown to improve statin-induced insulin resistance.^12^

**Figure 2.**
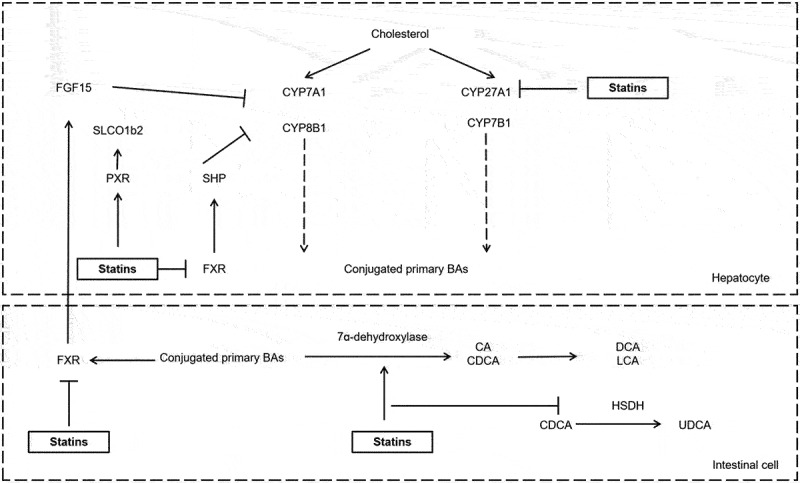
The mechanisms involved in statin metabolism. Statins influenced the FXR-SHP, FGF15, and PXR pathway.

Previous studies have identified that statins can affect the metabolism of TMAO, SFCAs, and BAs by regulating the microbiota. A meta-analysis found that statin treatment significantly decreased the TMAO levels, but there is a need for further evidence considering the types and doses of statins.^72^ Another study found that statin treatment resulted in a dramatic reduction in the production of butyric acid, whereas levels of acetic, propionic and valeric acids remained unaltered in both statin-treated and control groups.^46^ Growing evidence indicates that statin utilization is closely related to the bile acids metabolism. Statins have been reported to influence the farnesoid X receptor (FXR) pathway, a signaling pathway extensively involved in the regulation of bile acid and lipid metabolism. Fu et al. found that atorvastatin increased the mRNA levels of the BA-synthetic enzyme gene cholesterol 7α-hydroxylase (CYP7A1) by suppressing the expression of FXR target genes, called small heterodimer partner (SHP) and fibroblast growth factor 15 (FGF15).^73^ Besides, the hypolipidemic effect of Simvastatin by gut microbiota modulation was associated with the activation of CYP7A1, CYP7B1 and suppression of FXR.^31^ Rosuvastatin significantly reduced the hepatic expression of the Cytochrome P450 27A1 (CYP27A1), which is a key enzyme in the synthesis of cholic acid (CA) and chenodeoxycholic acid (CDCA).^49^ Statin affects the expression of PXR-targeted genes – SLCO1b2 (solute carrier organic anion transporter family, member 1b2), which encodes the major glucose transporter of the hepatocytes, and CYP3A11 (cytochrome P450, family 3, subfamily a, polypeptide 11), to be increased in atorvastatin-treated mice.^46^ Our recent study identified that atorvastatin reduced the level of 7-alpha-hydroxysteroid dehydrogenases (HSDH) and the conversion of CDCA to UDCA, resulting in insulin resistance^12^ (Figure 2, Table 1).

Statins affect enzymes in the secondary bile acid synthesis pathway. BA: bile acid, BSH: bile salt hydrolases, CA: cholic acid, CDCA: chenodeoxycholic acid, CYP7A1: cholesterol 7α-hydroxylase, CYP8B1: cholesterol 12-alpha-hydroxylase; CYP27A1: cytochrome P450 27A1, CYP7B1: cytochrome P450 Family 7 Subfamily B Member 1, DCA: deoxycholic acid, FXR: farnesoid X receptor, FGF15: fibroblast growth factor 15, HSDH: hydroxysteroid dehydrogenase, LCA: lithocholic acid, PXR: pregnane X receptor, SHP: small heterodimer partner, SLCO1b2: solute carrier organic anion transporter family, member 1b2, UDCA: ursodeoxycholic acid.

**Table 1.** Changes and mechanisms of statin-related gut microbiota or metabolites.

Study	Subject	Year	Changes of gut microbiota/metabolites			Mechanisms of statin	Reference
			Expression	Gut microbiota	Metabolites		
**Population cohort**	**Human**	**2018**	More	*Oscillospira*	—	Modulating the gut microbiota of the hypercholesterolemic patients	32
			Less	*Desulfovibrio*			
		**2018**	More	*Eubacterium, Faecalibacterium*	—	The gut microbiota is associated with the response to statin treatment	48
			Less	*Clostridium*			
		**2020**	More	—	—	Decreasing inflammation in patients with obesity-associated microbiota dysbiosis	35
			Less	*Bacteroides2* enterotype			
		**2021**	More	*Verrucomicrobia, Akkermansia, Lactobacillus*	—	The gut microbiota is associated with the response to statin treatment	37
			Less	*Holdemanella, Facecallibacterium*			
		**2021**	More	*Bifidobacterium, Anaerostipes, Ruminococcus*	—	Modulating the composition and function of the gut microbiome to reduce metabolic risk in patients with acute coronary syndrome	28
			Less	*Parabacteroides*			
		**2023**	More	—	—	Meta-analysis shows statins might reduce TMAO levels	72
			Less		TMAO		
		**2024**	More	*Clostridium sartagoforme*	—-	Changing the composition of the gut microbiota in statin users	47
			Less	*Bacteroides cellulosilyticus*			
**Animal experiment**	**Mice**	**2014**	More	—	Bile acids	Inducing bile acid-synthetic enzyme Cyp7a1 by suppressing FXR signaling	73
			Less				
		**2017**	More	*Proteobacteria*	Bile acids	Inducing bile acid-synthetic enzyme Cyp7a1 and Cyp7b1 by suppressing FXR signaling	31
			Less	*Firmicutes*			
		**2017**	More	*Bacteroidetes, Bacteroidales S24.7. group*	—	Activating the PXR pathway to affect the hepatic genes expression involved in lipid and glucose metabolism	46
			Less	*Firmicutes, Lachnospiraceae, Ruminococcaceae*	Butyrate		
		**2017**	More	*Rikenella, Coprococcus*	—	Decreasing the expression of TNF-α, IL-1β, NF-κB, and reducing levels of CA and CDCA	49
			Less	*Bilophila, Roseburia, Enterorhabdus, Akkermansia*	Bile acids		
		**2019**	More	*Bacteroides, Butyricimonas, Mucispirillum*	—	Decreasing IL-1β and increasing TGFβ1 to affect hyperglycemia and hyperlipidemia	33
			Less	—			
		**2020**	More	*Bacteroidetes*	—	Modulating the expression of Ldl-R, Srebp2 and Npc1l1	42，43
			Less	*Firmicutes*			
		**2022**	More	*Oscillibacter, Turicibacter, Anaerovorax, Parvibacter*	—-	Activating the intestinal NF-κB pathway and impairing the gut barrier function by suppressing Claudin1 levels	30
			Less	*Parabacteroides, Christensensellaces, Akkermansia muciniphila*			
		**2024**	More	*Barnesiella, Streptococcus*	CDCA	Resulting in insulin resistance and dysregulation of glucose homeostasis by suppressing the conversion of CDCA to UDCA and the expression of GLP-1	12
			Less	*Clostridium*	UDCA		

CA: cholic acid, CDCA: chenodeoxycholic acid, Cyp7a1: cholesterol 7α-hydroxylase, Cyp7b1: cytochrome P450 family 7 subfamily B member 1, GLP-1: glucagon-like peptide-1, IL-1β: interleukin-1β, Ldl-R: low-Density lipoprotein-receptor, NF-κB: nuclear factor-k-gene binding, Npc1l1: Niemann-Pick c1-like 1, TMAO: trimethylamine n-oxide, PXR: pregnane x receptor, Srebp2: sterol regulatory element-binding protein 2, TGFβ1: transforming growth factor-β, TNF-α: tumor necrosis factor-α, UDCA: ursodeoxycholic acid.

## 4. Gut microecology-mediated therapeutic effects and adverse reactions of statins

### 4.1. Gut microecology-mediated therapeutic effects after statin utilization

Since both statins and gut flora regulate lipid metabolism in the host, the effects of lipid-lowering drugs on the gut flora in mice were first explored by Emilie Catry et al. in 2015, who found that ezetimibe alone was able to increase the Lactobacillus spp.^74^ This provides us a new way of thinking about the important role of intestinal flora on statin efficacy based on the traditional lipid-lowering mechanism of statin. A subsequent study from China showed that hyperlipidemic patients who responded favorably to the lipid-lowering effects of atorvastatin (20 mg/d for 3 months) had a higher diversity of intestinal microbiota, including a greater abundance of *Lactobacillus, Eubacterium, Faecalibacterium, Bifidobacterium* and fewer *Clostridium* genera.^48^ Yinhui Liu et al. also found that the diversity of the gut microbiota was positively associated with the response to statin therapy (rosuvastatin 10 mg/d for 4–8 weeks) in hyperlipidemic patients.^75^ A recent case-crossover study reported that elevated serum cholesterol in statin users was associated with antibiotic use.^76^ In addition, Tomasz Wilmanski et al. found that a *Bacteroides*-enriched and diversity-poor gut microbiome was associated with stronger statin responses.^38^

In animal studies, Xuyun He et al. found that the LDL cholesterol-lowering effect of simvastatin disappeared following intestinal microecological dysregulation secondary to the administration of antibiotics.^31^ Our previous study also indicated that antibiotics weaken rosuvastatin’s lipid-lowering effect.^36^ Although differences in the type of statin, timing of intervention, and species across studies affect the modulation of the gut microbiota by statin, it is certain that the gut microbiota plays a key role in the efficacy of statin related lipid lowering.

**Figure 3.**
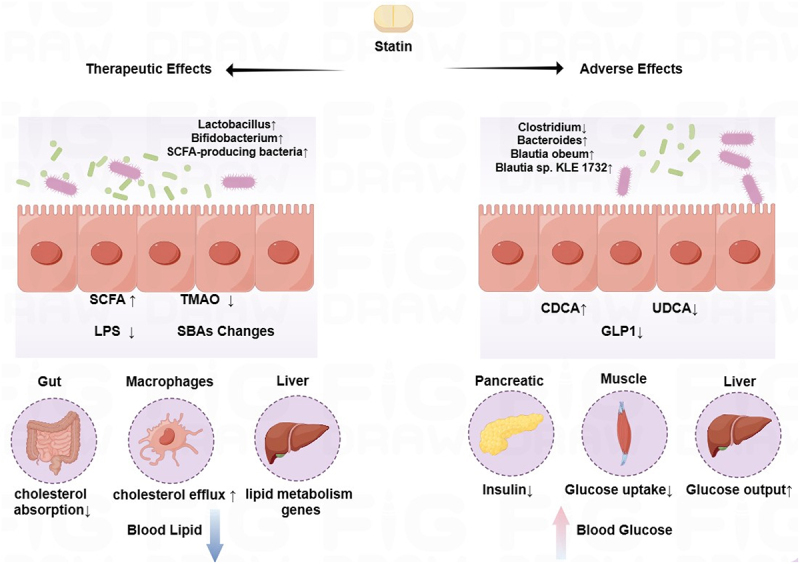
Efficacy and adverse effects of gut microbiota-mediated statins.

We propose that the gut microbiota can influence the lipid-lowering efficacy of statins in the following ways: (1) Gut microbiota alter statin metabolizing enzymes. Kaddurah-Daouk et al. found that secondary bile acids produced by gut microbes were associated with plasma levels of simvastatin and predicted the lipid-lowering effect of simvastatin, which may be an effect of the gut microbiota on the bioavailability of simvastatin.^77^ Dae-Hyoung Yoo et al. found that incubation of lovastatin with human and rat fecal enzyme preparations produced four metabolites, M1 (desmethyl butyryl metabolite), M4 (hydroxylated metabolite), M8 (reactive hydroxy acid metabolite), and M9 (hydroxylated M8), which suggested that the intestinal microbiota was involved in the metabolism of lovastatin. However, the formation of M8 in the feces of mice with the activity of lovastatin metabolizing enzymes was significantly decreased after the use of antibiotics.^39^ Thus, differences in individual gut microbiota may lead to differences in lipid-lowering efficacy by affecting the activity of statin-metabolizing enzymes. (2) Statin-induced changes in gut microbiota composition and microbiota-derived metabolites (MDMs) affect cholesterol absorption and accelerate cholesterol efflux. The increase in intestinal *Lactobacillus spp.* related to statin therapy has been widely reported.^40,41,48^ *Lactobacilli* strains have been reported to bind cholesterol to the cell surface and remove cholesterol from the culture medium, which may be related to their surface-secreted peptidoglycan and extracellular polysaccharide.^78^ Therefore, it is reasonable to hypothesize that statin leads to increased abundance of *Lactobacillus spp.* in the gut, thereby reducing intestinal cholesterol absorption. Previous studies have identified a higher SCFA-producing gut microbiota in atorvastatin-sensitive hyperlipidemia patients.^48^ Butyrate-producing gut microbiota were increased after statin treatment in animal studies.^33,79^ The effect of statin on the production of secondary bile acids (SBAs) by the gut microbiota has also been reported several times.^32,80^ It is reported that activation of FXR and TGR5 receptors as primary receptors for complex bile acid pools also inhibited intestinal cholesterol absorption and increased fecal cholesterol excretion.^78^ (3) MDMs indirectly influence statin’s lipid-lowering efficacy by regulating the expression of lipid metabolism genes in the liver. It is well known that the effect of statins on the gut microbiota will definitely lead to changes in MDMs. Statin was reported to decrease PPARγ, HMGCR, SREBP1, and FXR expression and increase CYP7A1 expression in the liver of HFD rats, and this effect was reversed after antibiotic therapy.^81^ Interestingly, Baolei Jia et al. summarized that SCFA and SBAs produced by the gut microbiota act on the same targets of statin regulation of hepatic lipid metabolism.^78^ Therefore, we speculated that MDMs might be a bridge linking statin and hepatic lipid metabolism-related genes. (4) Statin achieves lipid-lowering efficacy by reducing plasma TMAO levels and inhibiting LPS translocation^81,82^ (Figure 3).

Statin affects the secondary metabolites of the gut microbiota to produce a decrease in blood lipids and an increase in blood glucose. CDCA: Chenodeoxycholic Acid, GLP1: glucagon- like peptide-1, LPS: Lipopolysaccharides, SCFAs: Short-Chain Fatty Acids, SBAs: secondary bile acids, TMAO: Trimethylamine oxide, UDCA: ursodeoxycholic acid.

### 4.2. The role of gut microbiota in statin adverse reactions

Interactions between statin and gut microbiota may explain statin’s adverse effects. Although the adverse effects of statins are multifaceted, most of the current studies on gut microbiota focused on the adverse effects of statins on glucose metabolism. Many meta-analyses and observational data have shown that statin increases the risk of developing type 2 diabetes (T2DM).^83^ Researchers attributed this phenomenon to statin-induced weight gain and insulin resistance. However, slight weight gain is not enough to explain elevated blood glucose.

Emerging research has shown that the gut microbiota plays an important role in glucose metabolism. By retrospectively analyzing 42 studies on the relationship between gut microbiota and T2DM, Manoj Gurung et al. found that the genera of *Bifidobacterium, Bacteroides, Faecalibacterium, Akkermansia* and *Roseburia* were negatively associated with T2DM, while the genera of *Ruminococcus, Fusobacterium*, and *Blautia* were positively associated with T2DM.^84^ In addition, studies also revealed that secondary metabolites of the intestinal flora are important modulators in the course of T2DM. For example, SCFAs stimulated the secretion of intestinal peptide YY (PYY) and glucagon-like peptide-1 (GLP-1) from colonic L cells and protected intestinal mucosal integrity to prevent lipopolysaccharide (LPS) translocation.^85,86^ These studies give us hints that statin may adversely affect glucose metabolism by modulating the gut microbiota and its secondary metabolites.

It is found that statin therapy increased body weight and blood glucose levels in mice, which were mediated by profound changes in metabolic profiling of the gut flora composition.^29^ Another study reported that individuals with Bac.2 and Bac.1 intestinal types enriched in Bacteroides had the greatest interference with glycemic control with statins, whereas Rum. intestinal type appeared to be the most protective.^38^ Kari Koponenshouci reported for the first time that *Ruminococcus torques, Blautia obeum*, and *Blautia sp. KLE 1732* in the intestine were associated with an increased risk of statin-associated new-onset T2DM in an epidemiologic setting by large-sample population assessment using shotgun sequencing.^47^ However, none of the above studies revealed how gut microbiota specifically regulated glucose metabolism. Recently, we found that statins inhibited the conversion of CDCA to UDCA by decreasing the intestinal *Clostridium sp.* bacteria, causing decreased intestinal GLP-1 secretion, which leads to abnormal glucose metabolism and insulin resistance.^87^ Therefore, UDCA supplementation may be a therapeutic strategy to ameliorate the side effects of statin-induced glucose metabolism disruption. It is worth noting that the effects of statins on the gut microbiota are complex, and the gut flora itself can influence the drug metabolism of statins. The ultimate adverse effect of statins on glucose metabolism may be due to a stronger inhibitory effect on the pro-glucose metabolizing bacteria in the gut or a stronger promotional effect on the anti-glucose metabolizing bacteria.

There are few studies focusing on the role of the gut microbiota in statin-mediated adverse effects, so we are still unable to determine the specific mechanisms by which the gut microbiota is involved in other adverse effects of statin, such as myopathy or liver injury. However, we noted that a recent study showed that the majority (>90%) of muscle symptoms reported by all participants treated with statins were not due to statins.^88^ Therefore, it is still debatable whether myopathy is a side effect of statin. In conclusion, we expect more studies in the future to explore the role of gut microbiota in statin adverse effects (Figure 3).

### 4.3. Identification of gut microbiota markers that predict statin response or adverse events

The response and adverse effects of statins in different patients can be explained by changes in the human microbiome. Detection of the gut microbiome and appropriate interventions may increase the efficacy of statins and reduce the occurrence of adverse effects.

A study from China demonstrated that the increased genera *Lactobacillus*, *Eubacterium*, *Faecalibacterium,* and *Bifidobacterium*, and decreased genus *Clostridium* in patients may predict patient’s statin response and guide statin dosage adjustments.^48^ Another Chinese study reported that individuals dominated by *Bacteroides* have a better response to rosuvastatin.^75^ Tomasz Wilmanski et al. confirmed that a *Bacteroides*-enriched gut microbiome was associated with stronger statin responses.^38^ Conversely, individuals enriched in the *Ruminococcaceae* avoided the side effects of statins on insulin resistance, while also exhibiting a significant LDL-lowering response.^38^ Our previous study found that a poor response to statin treatment was associated with the decreased abundance of *Akkermansia muciniphila (A.* *muciniphila)* and increased abundance of *Lactobacillus, Holdemanella* and *Faecalibacterium.*^37^ Recently, we reported that reduction in *Clostridium sp.* was one of the mechanisms involved in statin-induced hyperglycemia.^87^

Combined with the above evidence, monitoring the composition of the gut microbiota may predict patient response to statins. Most studies have suggested that the abundance of *Lactobacillus* and *Bacteroides* is associated with stronger statin efficacy. Furthermore, *Clostridium sp.* appears to be the key gut microbe in this adverse effect of statin-induced hyperglycemia. Therefore, we hypothesized that monitoring of the above microbiota could be used to guide personalized statin regimens.

## 5. Individual variance in response to statin therapy

Although statins play a significant role in the treatment of cardiovascular diseases, interindividual variation in statin response is well known. The 2013 American College of Cardiology/American Heart Association guidelines define low -, medium -, and high-intensity statin therapy and the expected degree of LDL-C reduction.^89^ High-dose statins are expected to reduce LDL-C by at least 50%. However, treatment response is individual and varied, with a large proportion of individuals having less than 30% reduction in LDL-C.^90^ A meta-analysis involving 32,258 patients from 37 trials showed that the SD range of LDL-C reduction for all statin drugs and doses was 12.8% to 17.9%, and the percentage of patients with suboptimal reactions (LDL reduction <30%) ranged from 5.3% to 53.3%.^90^ This variability is not related to specific types or doses of statin drugs. In the JUPITER study, healthy participants with a median baseline LDL-C of 108 mg/dL receive rosuvastatin 20 mg. A 46% of the participants had a decrease of ≥50% in LDL-C, 43% had a decrease of 0–50% in LDL-C, and 11% had no decrease or increase in LDL-C compared to baseline.^91^

Variations in LDL-C response to statin therapy are attributed to phenotypic and genetic factors. Phenotypic predictors include age, ancestry, and smoking status.^92^ Genetic association studies have identified several single nucleotide polymorphisms (SNPs) and haplotypes associated with statin response.^93–97^ For example, candidate gene analysis found that variations in cholesterol metabolism regulators such as HMGCR, APOE, PCSK9, ACE, and LDLR were associated with statin response.^94–96^ In addition, genome-wide association studies (GWAS) have identified several SNPs at the LPA and APOE/TOMM40 sites, and their association with the magnitude of LDLC reduction has genome-wide significance.^97^ However, genetic factors associated with drug response or pharmacogenomics explained only a small fraction of drug variability. The statin-mediated LDLC reduction caused by these genotypes accounted for only about 4% of the overall difference.^97^ There is growing recognition that the microbiome may be an important underexplored factor leading to changes in drug metabolism and pharmacological efficacy.

A clinical study of 64 patients with hyperlipidemia who received rosuvastatin for 4–8 weeks was conducted to investigate the role of microbiota in mediating the lipid-lowering effect of rosuvastatin. This study found that changes in the community composition, taxonomic group, and diversity of gut microbiota are related to the response of statin to reduce LDL.^98^ Sun et al. assessed the bacterial composition and diversity of 202 patients with hyperlipidemia who were statin-sensitive or statin resistant. The results showed that statin-sensitive patients had higher intestinal biodiversity with increased *Lactobacillus, Eubacterium, Faecalibacterium* and *Bifidobacterium* genera and decreased *Clostridium* genera compared to statin-resistant group.^99^ Participants with a poor treatment response had significantly higher TMAO level than other participants who received placebo treatment.^34^ These results suggest that gut flora can directly or indirectly influence drug response by interfering with drug pharmacokinetics or pharmacodynamics. It is possible to modulate the gut microbiota for a better response to statins.

In addition to the difference in LDL-C response to statin therapy, other adverse effects were reported in patients treated with statins, such as statin-associated muscle symptoms (SAMS) and diabetes. Several factors may increase the risk of SAMS, including elderly, female, infirmity, high-dose statins, elevated serum concentrations of statin, hypothyroidism, the use of statin interacting drugs that inhibit statin catabolism, alcohol consumption, decreased muscle mass, and increased physical activity.^100–102^

The mechanism of new-onset diabetes after receiving statins is unknown, but statins may interfere with peripheral insulin signaling and pancreatic β cell function. In terms of genetic factors, a study showed that HMG-CoA inhibition was associated not only with lower plasma LDL-C levels, but also with a small increase of the body weight, waist circumference, plasma insulin and glucose concentrations.^103^ The data suggested that the increased risk of diabetes was partially due to the inhibition of HMG-CoA. A study published in 2017 evaluating the association between genetic variants of PCSK9 and T2DM showed that for every 1 mmol/l reduction in LDL-C levels, waist-to-hip ratio increased by 0.006, body weight increased by 1.02 kg, and fasting blood glucose levels increased by 0.009 mmol/l,^104^ which is consistent with HMCGR study. The odds ratio for newly diagnosed diabetes was 1.12.^104^ The data suggest that clinicians should screen diabetic patients for statin therapy and simultaneously initiate lifestyle change strategies.

Patients reveal a pronounced individual response to the administration of statins. Therefore, individual differences need to be considered in the design of statin treatment regimens to improve treatment outcomes and patient quality of life.

## 6. Future perspectives of statin intervention via gut microbiota modulation

The gut microbiota is vital for cardiovascular health. Gut microbiota produces various bioactive compounds including TMAO, SCFAs and bile acids, which help regulate blood lipids and glucose levels. Dysbiosis can harm the intestinal mucosa, facilitate bacterial and endotoxin entry into the bloodstream, and exacerbate inflammation. Statins are one of the most commonly used cardiovascular drugs. Recent studies have revealed a profound link between statin therapy and gut microbiota. Modulation of the gut microbiota and secondary metabolites may improve the effectiveness of statin therapy and reduce side effects.

Manipulation of the gut microbiota has great potential to improve both statin therapy and overall cardiovascular health. Recent research has suggested that changes in the gut microbiota may be associated with glucose metabolism disorders caused by statins.^12,35,38^ It was also reported that statins may affect the *Clostridium* species in the gut, reducing the conversion of CDCA to UDCA, thereby suppressing GLP-1 levels and leading to insulin resistance.^87^ As a result, it is also proposed that UDCA might be an effective therapeutic strategy among patients taking statins. By understanding the gut microbiota composition and metabolic characteristics of individuals, we will be able to provide more personalized medical treatment for patients who take statins as a routine for secondary prevention of cardiovascular diseases. But we also face many challenges that need to be addressed, including determining the appropriate dose and intervention regimen and implementing precise individual treatment. There is also a need to ensure that no serious adverse effects occur during the gut microbiota intervention.

In conclusion, considering the gut microbiota in personalized statin treatment strategies is crucial. Understanding how statin metabolism and efficacy are shaped by the gut microbiota can lead to more effective and safer statin therapy tailored to individual patients’ needs. For future researches, it is recommended to delve deeper into the gut microbiota’s role in statin metabolism and efficacy. Studies exploring the impact of dietary modifications, probiotics, or prebiotics on statin metabolism are warranted. Additionally, longitudinal studies tracking gut microbiota changes over time in statin users could provide valuable insights into long-term statin efficacy and tolerability.
